# *Camk2a-Cre* and *Tshz3* Expression in Mouse Striatal Cholinergic Interneurons: Implications for Autism Spectrum Disorder

**DOI:** 10.3389/fgene.2021.683959

**Published:** 2021-07-12

**Authors:** Xavier Caubit, Elise Arbeille, Dorian Chabbert, Florence Desprez, Imane Messak, Ahmed Fatmi, Bianca Habermann, Paolo Gubellini, Laurent Fasano

**Affiliations:** Aix-Marseille University, CNRS, IBDM, UMR 7288, Marseille, France

**Keywords:** *Camk2a-Cre*, *TSHZ3*, striatal cholinergic interneurons, autism spectrum disorder, *Mus musculus*

## Abstract

*Camk2a-Cre* mice have been widely used to study the postnatal function of several genes in forebrain projection neurons, including cortical projection neurons (CPNs) and striatal medium-sized spiny neurons (MSNs). We linked heterozygous deletion of *TSHZ3/Tshz3* gene to autism spectrum disorder (ASD) and used *Camk2a-Cre* mice to investigate the postnatal function of *Tshz3*, which is expressed by CPNs but not MSNs. Recently, single-cell transcriptomics of the adult mouse striatum revealed the expression of *Camk2a* in interneurons and showed *Tshz3* expression in striatal cholinergic interneurons (SCINs), which are attracting increasing interest in the field of ASD. These data and the phenotypic similarity between the mice with *Tshz3* haploinsufficiency and *Camk2a-Cre*-dependent conditional deletion of *Tshz3* (*Camk2a-cKO*) prompted us to better characterize the expression of *Tshz3* and the activity of *Camk2a-Cre* transgene in the striatum. Here, we show that the great majority of *Tshz3*-expressing cells are SCINs and that all SCINs express *Tshz3*. Using lineage tracing, we demonstrate that the *Camk2a-Cre* transgene is expressed in the SCIN lineage where it can efficiently elicit the deletion of the *Tshz3-*floxed allele. Moreover, transcriptomic and bioinformatic analysis in *Camk2a-cKO* mice showed dysregulated striatal expression of a number of genes, including genes whose human orthologues are associated with ASD and synaptic signaling. These findings identifying the expression of the *Camk2a-Cre* transgene in SCINs lineage lead to a reappraisal of the interpretation of experiments using *Camk2a-Cre*-dependent gene manipulations. They are also useful to decipher the cellular and molecular substrates of the ASD-related behavioral abnormalities observed in *Tshz3* mouse models.

## Introduction

The corticostriatal (CStr) circuitry is critically involved in functions ranging from motor control and habit formation to cognition. Defective development and dysfunction of CStr circuits have been linked to several brain disorders, including Huntington’s disease, Parkinson’s disease, and autism spectrum disorder (ASD) (Shepherd, 2013; Li and Pozzo-Miller, 2020). The striatum receives its main excitatory input from two types of cortical projection neurons (CPNs): intratelencephalic (IT) neurons and pyramidal-tract (PT) neurons. IT and PT neurons that project to the striatum reside mostly in the deep cortical layer (L) 5 (Reiner et al., 2010; Shepherd, 2013). Their primary striatal targets are the medium-sized spiny projection neurons (MSNs) (Sohur et al., 2012), which represent 90–95% of the neurons in the striatum (Gerfen and Surmeier, 2011). The remaining 5–10% of striatal neurons are aspiny interneurons, initially categorized into three main subpopulations, among which one population of striatal cholinergic interneurons (SCINs) (Kawaguchi, 1997). There is growing evidence for a higher diversity of striatal interneurons, based on studies on their developmental origin, gene expression profile, and electrophysiological properties and connectivity. These data define at least 6–7 classes of GABAergic interneurons (Munoz-Manchado et al., 2018; Tepper et al., 2018) and reveal that also SCINs are a heterogeneous population (Ahmed et al., 2019). Though few in number, striatal interneurons, and SCINs in particular, play a key functional role by modulating striatal activity through distinct connections with MSNs and/or other interneurons (Tepper et al., 2018; Abudukeyoumu et al., 2019).

We previously reported in mouse that heterozygous deletion of *Tshz3*, the gene encoding the transcription factor TSHZ3 (teashirt zinc-finger homeobox family member 3, also known as ZFP537), drives ASD-relevant behavioral abnormalities (Caubit et al., 2016), paralleled by altered transmission and plasticity at CStr synapses. Having shown that *Tshz3* is expressed within the CStr circuit in both L5 CPNs and a few striatal cells that are not MSNs (Caubit et al., 2016), we performed conditional *Tshz3* deletion using the *Camk2a-Cre* transgene, which is known to be active in CPNs from postnatal day 2–3 onward (Casanova et al., 2001; Li et al., 2017). We found that it results in a behavioral phenotype similar to *Tshz3* heterozygous mice, also associated with altered CStr function (Chabbert et al., 2019). These findings pointed to the CStr pathway as a main player in the ASD syndrome linked to *TSHZ3* deficiency. Recently, single-cell RNA sequencing (scRNA-seq) profiling showed that the striatal cells with the highest expression of *Tshz3* are SCINs: the levels of *Tshz3* transcripts in these cells are comparable to those found in CPNs, whereas they are very low in parvalbumin/tyrosine hydroxylase interneurons and almost null in the other striatal cell populations (Munoz-Manchado et al., 2018; Saunders et al., 2018). scRNA-seq data also revealed that the *Camk2a*, considered as being expressed in MSNs (von Schimmelmann et al., 2016; Andrade et al., 2017), is also expressed in subpopulations of striatal interneurons (Saunders et al., 2018). Given the phenotypic similarity between the heterozygous and conditional *Tshz3* mouse models, and the literature linking dysfunction of striatal circuits (Li and Pozzo-Miller, 2020) and altered cholinergic function to ASD (Karvat and Kimchi, 2014; Rapanelli et al., 2017), here we characterized in SCINs the expression of *Tshz3* and the activity of the *CamK2a-Cre* transgene using genetic lineage tracing (Heffner et al., 2012). Based on our results showing that the majority of SCINs express *Tshz3* and the *Camk2a-*Cre transgene, we characterized the molecular changes in the striatum resulting from *Tshz3* deletion using the *Camk2a-*Cre mice.

## Results

### The *Tshz3* Gene and the *Camk2a-Cre* Transgene Are Expressed in SCINs

**FIGURE 1:**
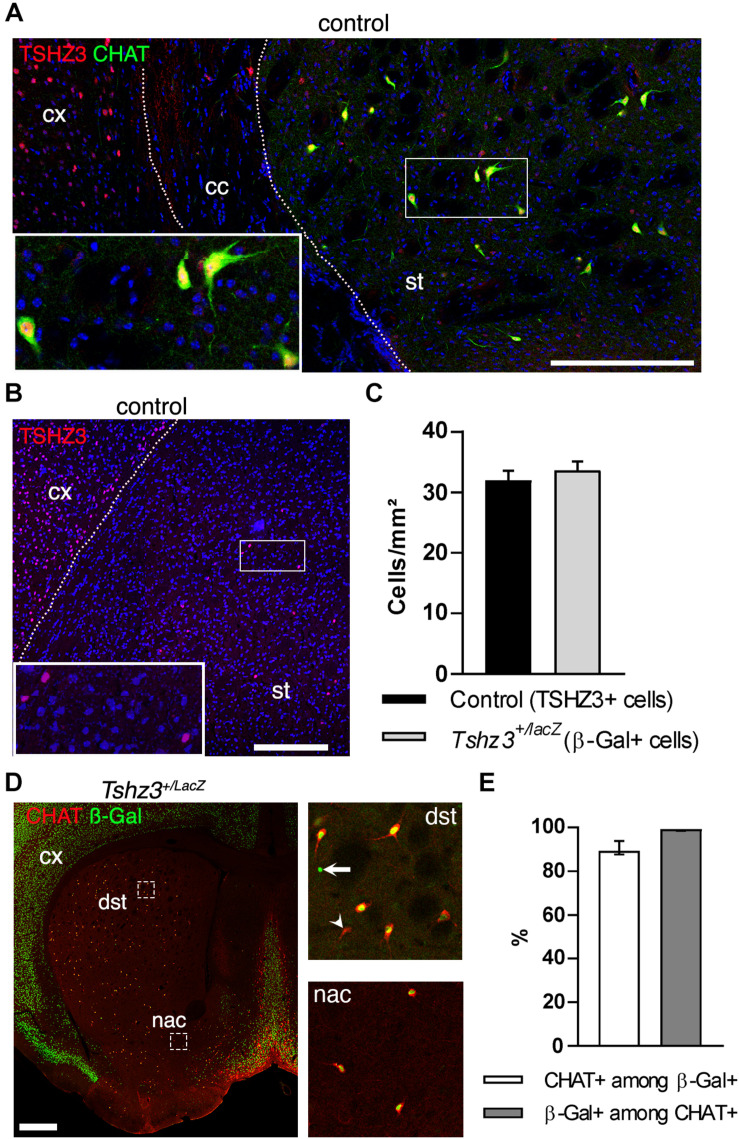
TSHZ3 is expressed in SCINs. **(A)** Immunostaining in a control mouse showing the expression of TSHZ3 and CHAT that identifies SCINs. Scale bar, 200 μm. **(B)** Immunostaining showing the expression of TSHZ3 in a control mouse. Scale bar, 250 μm. **(C)** Density of TSHZ3-positive (1791 cells in 56.0 mm^2^ from eight control mice) and ß-Gal-positive (954 cells in 28.1 mm^2^ from four *Tshz3^+/LacZ^* mice) striatal cells (*P* = 0.5225, Student’s *t*-test; data are expressed as mean + SEM). **(D)** Immunostaining showing the expression of ß-Gal (TSHZ3) and CHAT. Scale bar, 500 μm. Framed regions are shown on the right; arrow shows a ß-Gal-positive/CHAT-negative cell; arrowhead shows a CHAT-positive/ß-Gal-negative cell. **(E)** Percentage of CHAT-positive cells among ß-Gal-positive (TSHZ3+) cells, and vice-versa (12 sections from four *Tshz3^+/LacZ^* mice; data are expressed as median with interquartile range). cc, corpus callosum; cx, cerebral cortex; st, striatum. Nuclei in **A,B** are counterstained with DAPI (blue).

In the adult striatum, there are few TSHZ3-positive cells that are not MSNs (Caubit et al., 2016) and recent transcriptomic analysis of mouse striatum suggested *Tshz3* expression in SCINs (Munoz-Manchado et al., 2018; Saunders et al., 2018). To test this hypothesis, we performed double choline acetyltransferase (CHAT) and TSHZ3 immunohistochemistry in wild-type mice, confirming the expression of TSHZ3 in SCINs (Figure 1A). We also confirmed that, as reported in other tissues (Caubit et al., 2008), the expression of the *Tshz3*^*lacZ*^ allele in the striatum, revealed by beta-galactosidase (ß-Gal) staining, recapitulates the expression pattern of endogenous TSHZ3: accordingly, the density of TSHZ3-postive neurons in control mice is on the same range of the density of ß-Gal-positive neurons in *Tshz3^+/lacZ^* mice (Figures 1B–D). Moreover, these densities are in agreement with those reported in the literature (Matamales et al., 2016). Finally, double CHAT and ß-Gal staining in the striatum (Figures 1D,E) revealed that, in average, 90.4% of TSHZ3-expressing cells are SCINs and, conversely, 98.9% of SCINs co-express TSHZ3.

**FIGURE 2:**
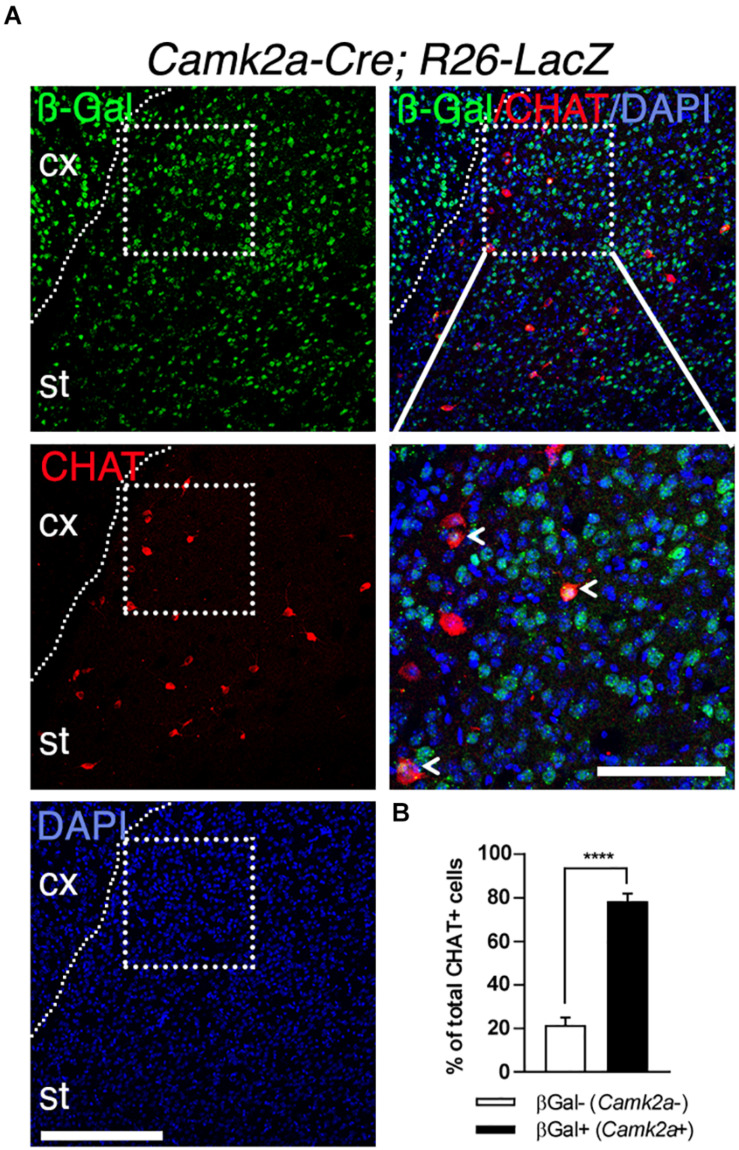
Expression of *Camk2a-Cre* in SCINs. **(A)** Coronal brain section from a *Camk2a-Cre;Rosa26-stop-LacZ* mouse stained for ß-Gal (TSHZ3) and CHAT. Scale bar, 200 μm. The boxed region is magnified to show ß-Gal/CHAT double positive SCINs (arrowheads). Scale bar, 100 μm. **(B)** Distribution of ß-Gal-negative and ß-Gal-positive cells among CHAT-positive cells (20 sections from three mice per genotype; ****P < 0.0001, Student’s *t*-test; data are expressed as means + SEM). cx, cerebral cortex; st, striatum. Nuclei were counterstained with DAPI (blue).

To further characterize the expression of the *Camk2a-Cre* transgene in the striatum, we performed a lineage tracing experiment by crossing *Camk2a-Cre* mice (Casanova et al., 2001) with *Rosa26-STOP-lacZ* mice (Mao et al., 1999). In these *Camk2a-Cre;Rosa26-STOP-lacZ* mice, the Cre activity resulted in the deletion of the STOP signal enabling *lacZ* expression in Cre-expressing cells and all their progeny. Accordingly, numerous ß-Gal-immunoreactive cells could be detected in the cerebral cortex and the striatum, consistent with *Camk2a* expression in CPNs and MSNs (Figure 2A). In addition, CHAT immunostaining showed that the majority (∼80%) of SCINs are also ß-Gal-positive (Figures 2A,B). Therefore, in the CStr circuit, *Tshz3* and *CamK2a* are co-expressed in CPNs and SCINs.

### The *Camk2a-Cre* Efficiently Deletes the *Tshz3*-Floxed Allele in SCINs

**FIGURE 3:**
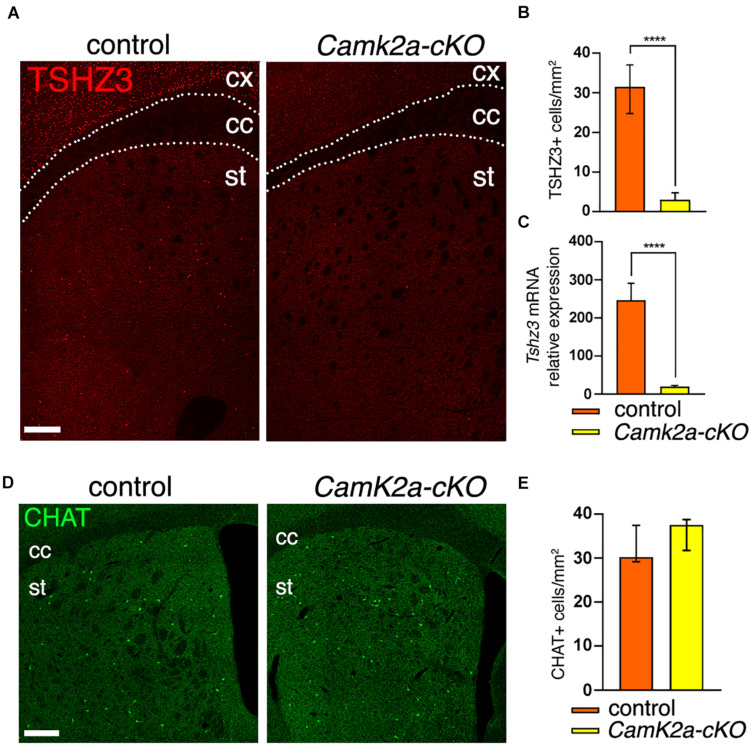
Loss of *Tshz3* in SCINs in *Camk2a-cKO* mice. **(A)** Coronal brain sections from control and *Camk2a-cKO* mice stained for TSHZ3. Scale bar, 500 μm. **(B)** Densities of TSHZ3-positive cells in the striatum of control and *Camk2a-cKO* mice (898 cells in 28.1 mm^2^ and 95 cells in 26.3 mm^2^ for control and *Camk2a-cKO*, respectively, from three mice per genotype; ****P < 0.0001, Mann–Whitney test). **(C)** *Tshz3* mRNA levels analyzed by RNA-seq in control and *Camk2a-cKO* striata (****P_*adj*_ < 1.00E-14, Log_2_FC = –2.26; data expressed as mean + SEM). **(D)** Coronal brain sections from control and *Camk2a-cKO* mice stained for CHAT. Scale bar, 300 μm **(E)** Densities of CHAT-positive SCINs in control and *Camk2a-cKO* mice (758 cells in 23.6 mm^2^ and 629 cells in 17.1 mm^2^ for control and *Camk2a-cKO*, respectively, from three mice per genotype; *P* = 0.1014, Mann–Whitney test). Cell counts in **B,E** were performed on the whole striatal surface. Data in **B,E** are expressed as median with interquartile range. cc, corpus callosum; cx, cerebral cortex; st, striatum.

We then examined whether *Camk2a-Cr*e expression by SCINs can drive the deletion of *Tshz3.* To address this issue, *Camk2a-Cr*e mice were crossed with *Tshz3*^*flox/flox*^ mice to obtain *Tshz3* conditional knock-out (*Camk2a-cKO*) mice. In this model we found that both the number of TSHZ3-positive cells (Figures 3A,B) and the levels of *Tshz3* mRNA (Figure 3C) in the striatum are dramatically reduced, while the density of CHAT-positive neurons is unchanged compared to control (Figures 3D,E). The remaining TSHZ3-positive cells do not exhibit a specific spatial distribution (data not shown).

### Genes Differentially Expressed in the Striatum of *Camk2a-cKO* Mice Are Associated With ASD

**FIGURE 4:**
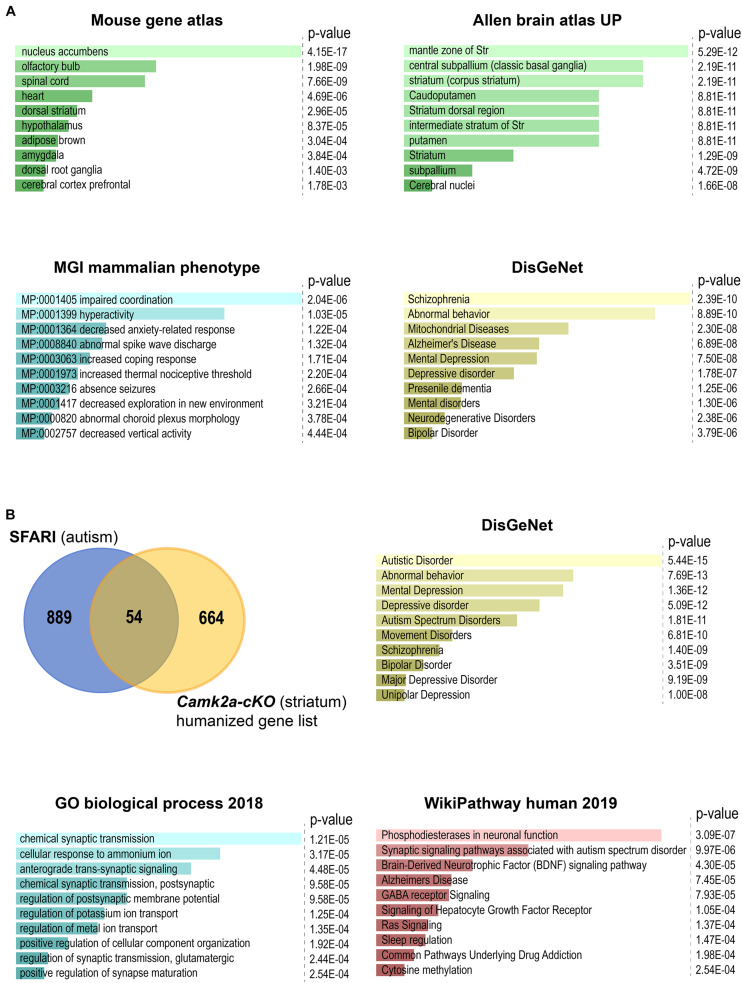
Enrichment analysis using EnrichR of DEGs between *Camk2a-cKO* and control. The bar plots show the top 10 terms selected according to their *P*-value. All enrichment results are also shown in Supplementary Tables 2, 3. **(A)** Analysis of all the DEGs. Highest-scoring is found for: nucleus accumbens, which is part of the ventral striatum, in the mouse gene atlas; terms related to striatum in the Allen brain atlas UP category; impaired coordination, which is commonly associated with ASD, in the MGI mammalian phenotype database; neurological disorders in the crowd-based DisGeNet. **(B)** Analysis of the 54 genes overlapping between the SFARI gene list and human orthologs of our list of DEGs. Highest scoring is found: in DisGeNet for autistic disorder (to note the strong overlap between these terms and those issued from the DisGeNet analysis of the overall set of DEGs); in Gene Ontology Biological Process for terms related to synaptic transmission and membrane potential; in human WikiPathways, for terms related to synaptic signaling pathways, including pathways associated with ASD.

Analysis of the RNA-seq data from the striatum of *Camk2a-cKO* mice (Supplementary Table 1A) revealed 210 differentially expressed genes (DEGs) with increased expression and 515 DEGs with decreased expression (false discovery rate cut-off = 0.075, log_2_ fold change > |0.25|) (Supplementary Table 1B). *Tshz3* itself is the most strongly downregulated gene. Comparison of the DEGs with scRNA-seq analysis of striatal interneurons (Munoz-Manchado et al., 2018) identified thirteen DEGs (*Bad, Dnpep, Fstl1, Galnt18, Hdac5, Id3, Mrpl54, Ncaph2, Ntrk1, Osbpl6, Sez6l, Tnrc18*, and *Tshz3*) expressed in SCINs. To further characterize the DEGs, we performed functional and enrichment analyses using EnrichR (Kuleshov et al., 2016) and g:Profiler (Raudvere et al., 2019). These analyses confirmed enrichment for genes expressed in the striatum (Supplementary Tables 1C–E) and revealed KEGG pathways mainly involved in mitochondrial function and neurological disorders (Figure 4A and Supplementary Tables 1F–H). We quantified the representation of 36 mitochondrial processes within the DEGs, using the MitoXplorer pipeline (Yim et al., 2020). This analysis revealed that DEGs are distributed in 31 mitochondrial processes, especially within oxidative phosphorylation, translation as well as replication and transcription (Supplementary Figure 1). The KEGG pathway enrichment analysis also identified the association of DEGs in three pathways (glutamatergic synapse, Wnt signaling, and mTOR signaling) associated with ASD (BRITE H02111) (Supplementary Tables 1F,G). We also performed enrichment analysis on phenotypes predefined by MGI (Mouse Genome Informatics mammalian phenotypes) and found that DEGs are significantly enriched with phenotypes of impaired coordination and hyperactivity (adjusted *P*-value < 0.05) (Supplementary Table 1I). Comparing the 718 human orthologs (Supplementary Table 1J) of the 725 DEGs with the SFARI autism gene list (Abrahams et al., 2013) identified 54 genes (Supplementary Table 1K). Almost half of these genes (25/54 = 46%) belong to the high confidence ASD candidates (SFARI categories 1 and 2). Amongst the ASD candidate gene set, the most significant KEGG pathways and biological processes are related to synaptic signaling (Figure 4B and Supplementary Table 2). We then proceeded to identify the DEGs present in both the striatum (725 DEGs; this study) and the cerebral cortex (1025 DEGs) (Chabbert et al., 2019) of *Camk2a-cKO* mice. This analysis identified 235 genes regulated in these two brain regions (Supplementary Table 3A), showing that most of the genes regulated by *Tshz3* are unique to the striatum or the cerebral cortex, suggesting cell-context specificity. Indeed, 196 genes regulated in both these brain structures are differentially responsive (activated in one brain part and repressed in the other, or vice versa), showing that even genes regulated by TSHZ3 both in the striatum and the cerebral cortex can be controlled by distinct mechanisms (Supplementary Table 3B). Comparing the 233 human orthologs (Supplementary Table 3C) of the 235 DEGs with SFARI identifies 15 genes including *TSHZ3* (Supplementary Table 3D). Last, functional and enrichment analysis using EnrichR reveals pathways involved in mitochondrial function and neurological disorders (Supplementary Tables 3E–H).

## Discussion

As main findings, this study show that (i) all SCINs express TSHZ3 and the main striatal population expressing TSHZ3 are SCINs; (ii) the *Camk2a-Cre* transgene is expressed in the SCIN lineage; (iii) conditional deletion of *Tshz3* using the *Camk2a-Cre* mediates efficient *Tshz3* deletion in SCINs and drives changes in the expression of 725 genes in the striatum, among which 196 are also differentially expressed in the cerebral cortex, suggesting profound alteration of striatal function. These results call to more caution in the interpretation of experiments using *Camk2a-Cre*-dependent gene manipulations and point to SCINs as potential players in the ASD-relevant behavioral abnormalities triggered by *Tshz3* loss in both the heterozygous deletion model (Caubit et al., 2016) and the conditional model using the *Camk2a-Cre* transgene (Chabbert et al., 2019).

Unreported recombinase activity using Cre driver lines can be due to ectopic expression of the *Cre* transgene but also to unappreciated expression of the driver gene itself (Heffner et al., 2012). The *Camk2a* gene has been reported to be expressed postnatally (Bayer et al., 1999) in specific brain regions, including the cerebral cortex and the striatum (Casanova et al., 2001). While the *Camk2a-Cre* transgene has been extensively used to study the postnatal function of genes highly expressed in projection neurons of these regions, as CPNs (Li et al., 2017) and MSNs (von Schimmelmann et al., 2016; Andrade et al., 2017), its expression and/or activity has not been specifically examined in striatal interneurons. To date, expression profiles available in gene expression atlas [i.e., Allen Brain Atlas (Lein et al., 2007)] do not provide information on the striatal cells expressing *CamK2a* and it is only recently that scRNA-seq reported the expression of *CamK2a* not only in MSNs but also in striatal interneurons (Saunders et al., 2018). In this context, genetic lineage tracing represents an efficient way to determine which cellular types express or have once expressed the *CamK2a-Cre* transgene whatever its developmental/postnatal expression profile. As MSNs represent 90% of all striatal neurons, expression of the reporter gene in a specific interneuron class, such as SCINs that constitute less than 2% of the total striatal population, cannot be addressed unless a specific marker for the latter is used. Here, we reveal the expression of the *Camk2a-Cre* transgene in the SCIN lineage by co-immunostaining for the reporter ß-Gal and for CHAT in the striatum of *Camk2a-Cre;Rosa26-STOP-lacZ* mice. The ß-Gal reporter is not detected in the whole SCIN population but in ∼80% of them. This could be due to methodological limitations, such as inefficient excision of the STOP cassette, and/or reflect the diversity of these interneurons recently emphasized by studies of their developmental origin and birthdate (Allaway and Machold, 2017), molecular and electrophysiological profile, and connectivity (Ahmed et al., 2019). In addition, our results call for a better characterization of the expression pattern of the *CamK2a-Cre* transgene, critical for the interpretation of the results generated using this Cre line. In particular, they raise the question of the contribution of SCINs to the phenotypes observed in mouse models of *Tshz3*-deficiency. For instance, we show that the *Tshz3* gene associated with autism (Caubit et al., 2016; Chabbert et al., 2019), known to be expressed in CPNs, is also expressed in SCINs. Moreover, expression of *Cre* allows efficient recombination of the *Tshz3*-floxed allele in SCINs without affecting their viability, as previously shown for CPNs. Therefore, *Tshz3* deletion in both CPNs and SCINs might contribute to altered functioning of the CStr circuitry in the heterozygous *Tshz3* as well as in the conditional *Camk2a-cKO* mouse models. It is worth noting that, although in low number, SCINs are tonically active neurons that represent the main source of striatal cholinergic tone, and act as key regulators of striatal function in health and diseases. Despite evidence for the role of the cholinergic system in the etiology of ASD (Karvat and Kimchi, 2014), the specific involvement of SCINs remains poorly investigated. The findings of this study raise the question of the respective contribution of SCINs and CPNs to the *TSHZ3*-related ASD phenotype.

In addition, we found 725 DEGs in the striatum of *Camk2a-cKO* mice. These gene expression changes might occur in part in SCINs, in particular for the DEGs reported as specific for these interneurons (Munoz-Manchado et al., 2018) as well as in the few *Tshz3*-positive/CHAT-negative striatal cells, in CPN axons (Kim and Jung, 2020) and, in a non-cell autonomous way, in other striatal components. Indeed, we previously reported that *Tshz3* deletion results in altered transmission and plasticity at corticostriatal synapses (Chabbert et al., 2019), which could in turn affect gene expression in their striatal targets (mainly MSNs). *Tshz3* loss in SCINs might also alter their morphofunctional properties and thereby indirectly impact gene expression in their striatal targets. Regarding the pathways dysregulated in the striatum of *Camk2a-cKO* mice, enrichment analysis highlights synaptic activity and mitochondrial function pathways, whose alterations are suggested to contribute to ASD development (Citrigno et al., 2020; Rojas-Charry et al., 2021). In the same line, testing for enrichment of MGI mammalian phenotypes associated with the DEGs identified impaired coordination and hyperactivity, two behaviors that frequently accompany ASD. Similarly, enrichment for Elsevier pathway collection identified DEGs involved in epilepsy, which is found at higher rates in children with ASD than the general population. In conclusion, here we show that conditional *Tshz3* deletion using the *Camk2a-Cre* transgene targets not only CPNs (Casanova et al., 2001; Li et al., 2017) but, unexpectedly, also SCINs, triggering dramatic changes in striatal gene expression. These data call for possible reconsideration of previous findings obtained using *Camk2a-Cr*e-dependent gene deletion, and for careful characterization of transgenic Cre mouse lines.

## Materials and Methods

### Mouse Strains and Genotyping

The *Tshz3^*lacZ*^, Tshz3*^*flox/flox*^, *Camk2a-Cre* [*CaMK2a-iCre* BAC (CKC)] and *Rosa26-STOP-lacZ* mouse lines have been described previously (Mao et al., 1999; Casanova et al., 2001; Caubit et al., 2008; Chabbert et al., 2019). Male heterozygous *Camk2a-Cre* mice were crossed with female *Tshz3*^*flox/flox*^ to obtain *Tshz3* conditional knock-out (cKO) mice (*Camk2a-cKO*) (Casanova et al., 2001). Littermate *Camk2a-Cre^–/–^* mice were used as control. Animals carrying the *Tshz3*^*flox*^ allele and *Tshz3*^Δ^ allele were genotyped as described previously (Chabbert et al., 2019).

### Immunohistochemistry and Histology

All stains were performed on coronal 40 μm cryostat brain sections of postnatal day (P) 28–34 mice, cut at the level of the rostral striatum, from bregma 0 to +1.18 mm, AP (Paxinos and Franklin, 2001). For TSHZ3 immunostaining, brains were immediately removed after euthanasia and frozen in dry ice until use; before incubation with the antibodies, sections were fixed in 4% paraformaldehyde (PFA) for 15 min, then washed twice for 5 min in PBS. For TSHZ3 and CHAT double immunodetection, mice were anesthetized (ketamine + xylazine, 100 + 10 mg/kg, respectively, i.p.) and transcardially perfused with PBS. Brains were immediately dissected out, post-fixed by immersion 2 h in 4% paraformaldehyde in PBS, placed in 30% sucrose in PBS overnight and frozen in dry ice until sectioning. For the other stains, mice under anesthesia were transcardially perfused with 4% PFA in PBS. Brains were removed and post-fixed in 4% PFA for at least 2 h before cryostat sectioning. For all stains, brain sections were washed with PBS and blocked in PBST (0.3% Triton X-100 in 1xPBS) with 5% BSA for 1 h at room temperature. Sections were then incubated in primary antibody diluted in blocking solution (PBST, 1% BSA) overnight at 4°C with the following primary antibodies: goat anti-CHAT (1:100, Millipore, AB144P), rabbit anti-ß-Galactosidase (1:1,000, Cappel, 599762) and guinea-pig anti-TSHZ3 (1:2,000; ref. Caubit et al., 2008). Sections were then washed with PBS three times and incubated overnight at 4°C in secondary antibodies diluted 1:1,000 in blocking solution: donkey anti-guinea pig Cy3 and donkey anti-goat Cy3 (Jackson ImmunoResearch Laboratories); donkey anti-goat Alexa Fluor 488, Donkey anti-Goat AlexaFluor 568 and donkey anti-rabbit Alexa Fluor 488 (Life Technologies). Sections were stained using a 300 μM DAPI intermediate solution (1:1,000, Molecular Probes, Cat# B34650). Sections were then washed with PBS three times, mounted on Superfrost Plus slides (Fischer Scientific) and coverslipped for imaging on a laser scanning confocal microscope (Zeiss LSM780 with Quasar detection module). Spectral detection bandwidths (nm) were set at 411-473 for DAPI, 498-568 for GFP and 568-638 for Cy3; pinhole was set to 1 Airy unit. Unbiased counting of CHAT-, TSHZ3-, and ß-Gal-positive neurons were done on the whole surface the dorsal striatum (excluding the nucleus accumbens) of confocal images using ImageJ software (see figure legends for details). Images were assembled using Photoshop 21.2.3. Statistical analysis was performed using GraphPad Prism 7.05. Data were analyzed by unpaired Student’s *t*-test or by Mann–Whitney test when they passed or not, respectively, the D’Agostino–Pearson normality test. A *P*-value < 0.001 was considered significant.

### RNA Sequencing Analysis

Three independent replicates, each containing the dorsal striata from 3 to 4 *Camk2a-cKO* mice and littermate controls (P34), were prepared for analysis. RNA and cDNA preparation, as well as cDNA sequencing, were performed as previously reported (Chabbert et al., 2019). We used STAR (Dobin et al., 2013) with standard parameters to align RNA-seq reads to the latest release of the mouse genome (mm10) downloaded from UCSC (as of March 2020). Read counting was performed using featureCounts (Liao et al., 2014). One replicate of control and one of *Camk2a-cKO* were discarded before further analysis due to inconsistent clustering during principal component analysis. Differential expression analysis was done using DESeq2 (Love et al., 2014). DESeq2 results, as well as the list of DEGs used for enrichment analysis and KEGG pathway mapping (representing genes with a FDR ≤ 0.075 and a fold change of 1.25 (log2FC: |0.25|), are shown in Supplementary Table 1B. Enrichment analysis was done using EnrichR (Chen et al., 2013; Kuleshov et al., 2016).

### Quantitative RT-qPCR

Total RNA from control and *Tshz3* mutant (P28) cerebral cortex was prepared using Rneasy Plus Universal Mini Kit gDNA eliminator (Qiagen^TM^) and first strand cDNA was synthesized using iScript Reverse Transcription Supermix kit (Bio-RAD^TM^). Real-time quantitative PCR (RT-qPCR) was performed on a CFX96 qPCR detection system (Bio-RAD^TM^) using SYBR^®^ GreenER^TM^ qPCR SuperMixes (Life Technologies^TM^). RT-qPCR conditions: 40 cycles of 95°C for 15 s and 60°C for 60 s. Analyses were performed in triplicate. Transcript levels were first normalized to the housekeeping gene *Gapdh*. Primer sequences used for RT-qPCR: *Gapdh* Forward: 5′-GTCTCCTGCGACTTCAACAGCA-3′; *Gapdh* Reverse: 5′-ACCACCCTGTTGCTGTAGCCGT-3′. *Tshz3* Forward: 5′-CACTCCTTCCAGCATCTCTGAG-3′; *Tshz3* Reverse: 5′-TAGCAGGTGCTGAGGATTCCAG-3′. Statistical analysis was performed by unpaired Student’s *t*-tests by using the qbasePLUS software version 2 (Biogazelle). A *P*-value < 0.05 was considered significant.

## URLs

http://dropviz.org

https://www.genome.jp/kegg/brite.html

http://mitoxplorer.ibdm.univ-mrs.fr.

## Data Availability Statement

The data that support the findings of this study are available from the corresponding author upon reasonable request. Raw data (FastQ files) from the sequencing experiment (triplicates from wild-type and *Tshz3*-mutant striatum) and raw abundance measurements for genes (read counts) for each sample are available from Gene Expression Omnibus (GEO) under accession GSE157658, which should be quoted in any manuscript discussing the data.

## Ethics Statement

The animal study was reviewed and approved by “Comité National de Réflexion Ethique sur l’Expérimentation Animale n°14” (ID numbers 57-07112012, 2019020811238253-V2 #19022, and 2020031615241974-V5 #25232) and were in agreement with the recommendations of the European Communities Council Directive (2010/63/EU).

## Author Contributions

EA, XC, DC, and FD performed the histological experiments and quantitative analyses. EA, XC, and FD performed the CHAT cell lineage experiments. AF and XC prepared the samples for RNA-seq. BH and IM performed the RNA-seq analysis, pathway analysis, and enrichment analysis. EA and XC generated and maintained the transgenic mouse lines. XC, LF, BH, and PG conceived the project, supervised the work and wrote the manuscript. All authors contributed to the article and approved the submitted version.

## Conflict of Interest

The authors declare that the research was conducted in the absence of any commercial or financial relationships that could be construed as a potential conflict of interest.

## Supplementary Material

Supplementary Figure 1 Interactome view of 36 mitochondrial processes within the DEGs. Striatal DEGs are distributed in 31 mitochondrial processes, especially within oxidative phosphorylation, translation as well as replication and transcription. Individual bubbles represent genes of a process; blue and red, indicate up- or down-regulated genes within a process, respectively. Bubble size indicates log2 fold change values. This scheme was built using the “Interactome View” tool available on mitoXplorer web-plateform http://mitoxplorer.ibdm.univ-mrs.fr/index.php.

## References

[B1] Abrahams B. S. Arking D. E. Campbell D. B. Mefford H. C. Morrow E. M. Weiss L. A. (2013). Sfari gene 2.0: a community-driven knowledgebase for the autism spectrum disorders (asds). *Mol. Autism* 4:36. 10.1186/2040-2392-4-36 24090431 PMC3851189

[B2] Abudukeyoumu N. Hernandez-Flores T. Garcia-Munoz M. Arbuthnott G. W. (2019). Cholinergic modulation of striatal microcircuits. *Eur. J. Neurosci.* 49 604–622. 10.1111/ejn.13949 29797362 PMC6587740

[B3] Ahmed N. Y. Knowles R. Dehorter N. (2019). New insights into cholinergic neuron diversity. *Front. Mol. Neurosci.* 12:204. 10.3389/fnmol.2019.00204 31551706 PMC6736589

[B4] Allaway K. C. Machold R. (2017). Developmental specification of forebrain cholinergic neurons. *Dev. Biol.* 421 1–7. 10.1016/j.ydbio.2016.11.007 27847324

[B5] Andrade E. C. Musante V. Horiuchi A. Matsuzaki H. Brody A. H. Wu T. (2017). Arpp-16 is a striatal-enriched inhibitor of protein phosphatase 2a regulated by microtubule-associated serine/threonine kinase 3 (mast 3 kinase). *J. Neurosci.* 37 2709–2722. 10.1523/JNEUROSCI.4559-15.2017 28167675 PMC5354324

[B6] Bayer K. U. Lohler J. Schulman H. Harbers K. (1999). Developmental expression of the cam kinase ii isoforms: ubiquitous gamma- and delta-cam kinase ii are the early isoforms and most abundant in the developing nervous system. *Brain Res. Mol. Brain Res.* 70 147–154. 10.1016/s0169-328x(99)00131-x 10381553

[B7] Casanova E. Fehsenfeld S. Mantamadiotis T. Lemberger T. Greiner E. Stewart A. F. (2001). A camkiialpha icre bac allows brain-specific gene inactivation. *Genesis* 31 37–42. 11668676 10.1002/gene.1078

[B8] Caubit X. Gubellini P. Andrieux J. Roubertoux P. L. Metwaly M. Jacq B. (2016). Tshz3 deletion causes an autism syndrome and defects in cortical projection neurons. *Nat. Genet.* 48 1359–1369. 10.1038/ng.3681 27668656 PMC5083212

[B9] Caubit X. Lye C. M. Martin E. Core N. Long D. A. Vola C. (2008). Teashirt 3 is necessary for ureteral smooth muscle differentiation downstream of shh and bmp4. *Development* 135 3301–3310. 18776146 10.1242/dev.022442

[B10] Chabbert D. Caubit X. Roubertoux P. L. Carlier M. Habermann B. Jacq B. (2019). Postnatal tshz3 deletion drives altered corticostriatal function and autism spectrum disorder-like behavior. *Biol. Psychiatry* 86 274–285. 10.1016/j.biopsych.2019.03.974 31060802

[B11] Chen E. Y. Tan C. M. Kou Y. Duan Q. Wang Z. Meirelles G. V. (2013). Enrichr: interactive and collaborative html5 gene list enrichment analysis tool. *BMC Bioinform.* 14:128. 10.1186/1471-2105-14-128 23586463 PMC3637064

[B12] Citrigno L. Muglia M. Qualtieri A. Spadafora P. Cavalcanti F. Pioggia G. (2020). The mitochondrial dysfunction hypothesis in autism spectrum disorders: current status and future perspectives. *Int. J. Mol. Sci.* 21:5785. 10.3390/ijms21165785 32806635 PMC7461038

[B13] Dobin A. Davis C. A. Schlesinger F. Drenkow J. Zaleski C. Jha S. (2013). Star: ultrafast universal rna-seq aligner. *Bioinformatics* 29 15–21. 10.1093/bioinformatics/bts635 23104886 PMC3530905

[B14] Gerfen C. R. Surmeier D. J. (2011). Modulation of striatal projection systems by dopamine. *Annu. Rev. Neurosci.* 34 441–466. 10.1146/annurev-neuro-061010-113641 21469956 PMC3487690

[B15] Heffner C. S. Herbert Pratt C. Babiuk R. P. Sharma Y. Rockwood S. F. Donahue L. R. (2012). Supporting conditional mouse mutagenesis with a comprehensive cre characterization resource. *Nat. Commun.* 3:1218. 10.1038/ncomms2186 23169059 PMC3514490

[B16] Karvat G. Kimchi T. (2014). Acetylcholine elevation relieves cognitive rigidity and social deficiency in a mouse model of autism. *Neuropsychopharmacology* 39 831–840. 10.1038/npp.2013.274 24096295 PMC3924518

[B17] Kawaguchi Y. (1997). Neostriatal cell subtypes and their functional roles. *Neurosci. Res.* 27 1–8. 10.1016/s0168-0102(96)01134-0 9089693

[B18] Kim E. Jung H. (2020). Local mrna translation in long-term maintenance of axon health and function. *Curr. Opin. Neurobiol.* 63 15–22. 10.1016/j.conb.2020.01.006 32087477

[B19] Kuleshov M. V. Jones M. R. Rouillard A. D. Fernandez N. F. Duan Q. Wang Z. (2016). Enrichr: a comprehensive gene set enrichment analysis web server 2016 update. *Nucleic Acids Res.* 44 W90–W97. 10.1093/nar/gkw377 27141961 PMC4987924

[B20] Lein E. S. Hawrylycz M. J. Ao N. Ayres M. Bensinger A. Bernard A. (2007). Genome-wide atlas of gene expression in the adult mouse brain. *Nature* 445 168–176. 10.1038/nature05453 17151600

[B21] Li W. Pozzo-Miller L. (2020). Dysfunction of the corticostriatal pathway in autism spectrum disorders. *J. Neurosci. Res.* 98 2130–2147. 10.1002/jnr.24560 31758607 PMC7242149

[B22] Li Y. You Q. L. Zhang S. R. Huang W. Y. Zou W. J. Jie W. (2017). Satb2 ablation impairs hippocampus-based long-term spatial memory and short-term working memory and immediate early genes (iegs)-mediated hippocampal synaptic plasticity. *Mol. Neurobiol.* 10.1007/s12035-017-0531-5 [Epub ahead of print]. 28421537

[B23] Liao Y. Smyth G. K. Shi W. (2014). Featurecounts: an efficient general purpose program for assigning sequence reads to genomic features. *Bioinformatics* 30 923–930. 10.1093/bioinformatics/btt656 24227677

[B24] Love M. I. Huber W. Anders S. (2014). Moderated estimation of fold change and dispersion for rna-seq data with deseq2. *Genome Biol.* 15:550. 10.1186/s13059-014-0550-8 25516281 PMC4302049

[B25] Mao X. Fujiwara Y. Orkin S. H. (1999). Improved reporter strain for monitoring cre recombinase-mediated DNA excisions in mice. *Proc. Natl. Acad. Sci. U. S. A.* 96 5037–5042. 10.1073/pnas.96.9.5037 10220414 PMC21812

[B26] Matamales M. Gotz J. Bertran-Gonzalez J. (2016). Quantitative imaging of cholinergic interneurons reveals a distinctive spatial organization and a functional gradient across the mouse striatum. *PLoS One* 11:e0157682. 10.1371/journal.pone.0157682 27314496 PMC4912095

[B27] Munoz-Manchado A. B. Bengtsson Gonzales C. Zeisel A. Munguba H. Bekkouche B. Skene N. G. (2018). Diversity of interneurons in the dorsal striatum revealed by single-cell rna sequencing and patchseq. *Cell Rep.* 24 2179–2190.e7. 10.1016/j.celrep.2018.07.053 30134177 PMC6117871

[B28] Paxinos G. Franklin K. B. J. (2001). *The Mouse Brain in Stereotaxic Coordinates*, 2nd Edn. Cambridge, MA: Academic Press.

[B29] Rapanelli M. Frick L. R. Xu M. Groman S. M. Jindachomthong K. Tamamaki N. (2017). Targeted interneuron depletion in the dorsal striatum produces autism-like behavioral abnormalities in male but not female mice. *Biol. Psychiatry* 82 194–203. 10.1016/j.biopsych.2017.01.020 28347488 PMC5374721

[B30] Raudvere U. Kolberg L. Kuzmin I. Arak T. Adler P. Peterson H. (2019). G:Profiler: a web server for functional enrichment analysis and conversions of gene lists (2019 update). *Nucleic Acids Res.* 47 W191–W198. 10.1093/nar/gkz369 31066453 PMC6602461

[B31] Reiner A. Hart N. M. Lei W. Deng Y. (2010). Corticostriatal projection neurons – dichotomous types and dichotomous functions. *Front. Neuroanat.* 4:142. 10.3389/fnana.2010.00142 21088706 PMC2982718

[B32] Rojas-Charry L. Nardi L. Methner A. Schmeisser M. J. (2021). Abnormalities of synaptic mitochondria in autism spectrum disorder and related neurodevelopmental disorders. *J. Mol. Med. (Berl.)* 99 161–178. 10.1007/s00109-020-02018-2 33340060 PMC7819932

[B33] Saunders A. Macosko E. Z. Wysoker A. Goldman M. Krienen F. M. de Rivera H. (2018). Molecular diversity and specializations among the cells of the adult mouse brain. *Cell* 174 1015–1030e1016. 10.1016/j.cell.2018.07.028 30096299 PMC6447408

[B34] Shepherd G. M. (2013). Corticostriatal connectivity and its role in disease. *Nat. Rev. Neurosci.* 14 278–291. 10.1038/nrn3469 23511908 PMC4096337

[B35] Sohur U. S. Padmanabhan H. K. Kotchetkov I. S. Menezes J. R. Macklis J. D. (2012). Anatomic and molecular development of corticostriatal projection neurons in mice. *Cereb. Cortex* 24 293–303. 10.1093/cercor/bhs342 23118198 PMC3888374

[B36] Tepper J. M. Koos T. Ibanez-Sandoval O. Tecuapetla F. Faust T. W. Assous M. (2018). Heterogeneity and diversity of striatal gabaergic interneurons: update 2018. *Front. Neuroanat.* 12:91. 10.3389/fnana.2018.00091 30467465 PMC6235948

[B37] von Schimmelmann M. Feinberg P. A. Sullivan J. M. Ku S. M. Badimon A. Duff M. K. (2016). Polycomb repressive complex 2 (prc2) silences genes responsible for neurodegeneration. *Nat. Neurosci.* 19 1321–1330. 10.1038/nn.4360 27526204 PMC5088783

[B38] Yim A. Koti P. Bonnard A. Marchiano F. Durrbaum M. Garcia-Perez C. (2020). Mitoxplorer, a visual data mining platform to systematically analyze and visualize mitochondrial expression dynamics and mutations. *Nucleic Acids Res.* 48 605–632. 10.1093/nar/gkz1128 31799603 PMC6954439

