# Supplementary material for: Camk2a-Cre and Tshz3 Expression in Mouse Striatal Cholinergic Interneurons: Implications for Autism Spectrum Disorder
Source: Front Genet. 2021 Jul 12;12:683959. doi: 10.3389/fgene.2021.683959 (PMC8328143; doi:10.3389/fgene.2021.683959)
Supplement: Supplementary Figure 1 — Interactome view of 36 mitochondrial processes within the DEGs. Striatal DEGs are distributed in 31 mitochondrial processes, especially within oxidative phosphorylation, translation as well as replication and transcription. Individual bubbles represent genes of a process; blue and red, indicate up- or down-regulated genes within a process, respectively. Bubble size indicates log2 fold change values. This scheme was built using the “Interactome View” tool available on mitoXplorer web-plateform http://mitoxplorer.ibdm.univ-mrs.fr/index.php. [file Data_Sheet_1.pdf]

Supplementary Material

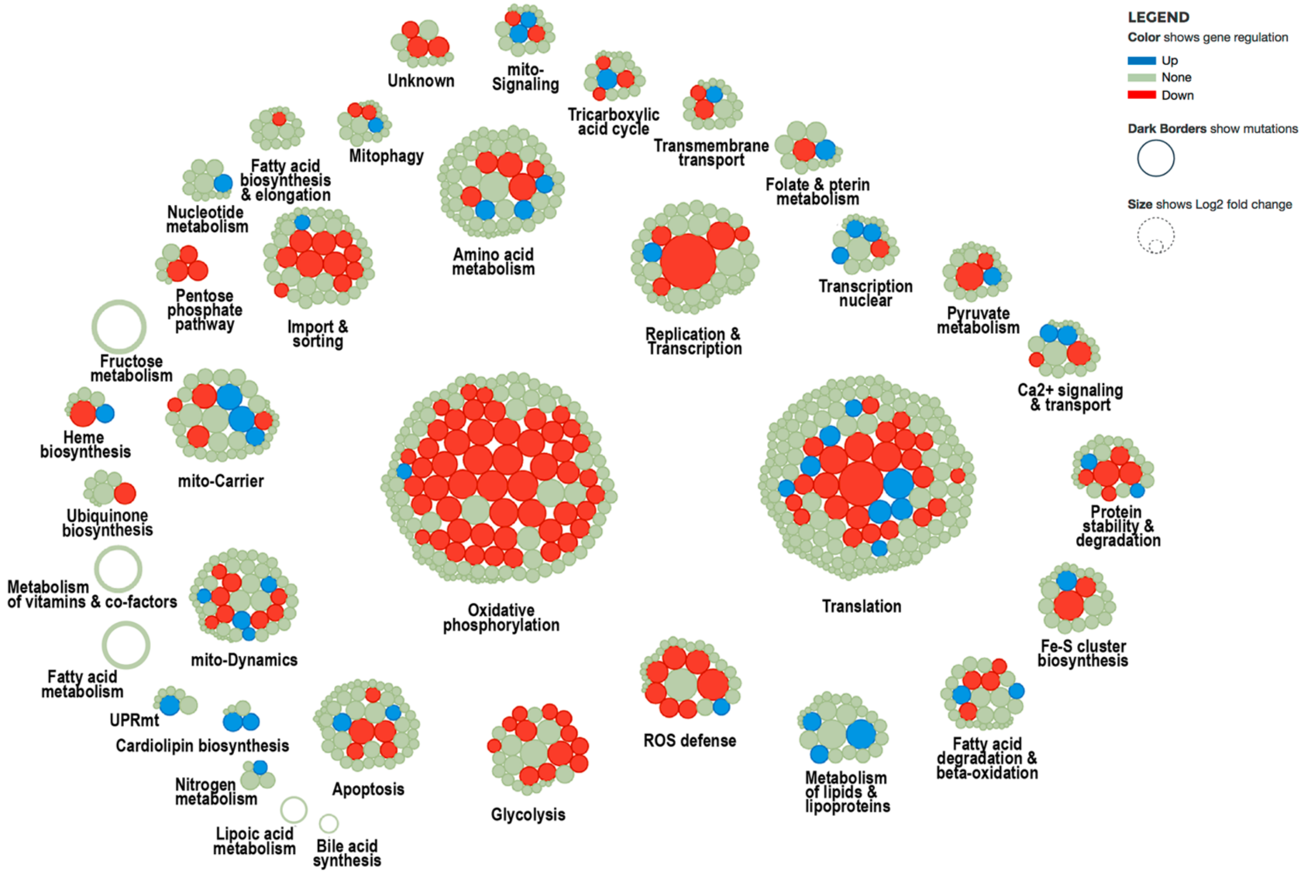

Supplementary Figure 1. Interactome view of 36 mitochondrial processes within the DEGs. Striatal DEGs are distributed in 31 mitochondrial processes, especially within oxidative phosphorylation, translation as well as replication and transcription. Individual bubbles represent genes of a process; blue and red, indicate up- or down-regulated genes within a process, respectively. Bubble size indicates log2 fold change values. This scheme was built using the “Interactome View” tool available on mitoXplorer web-platform http://mitoxplorer.ibdm.univ-mrs.fr/index.php .
